# Butanol production from lignocellulosic sugars by *Clostridium beijerinckii* in microbioreactors

**DOI:** 10.1186/s13068-021-01886-1

**Published:** 2021-01-30

**Authors:** Cansu Birgen, Kristin F. Degnes, Sidsel Markussen, Alexander Wentzel, Håvard Sletta

**Affiliations:** 1 Department of Chemical Engineering, NTNU, 7491 Trondheim, Norway; 2 Department of Thermal Energy, SINTEF Energy Research, 7034 Trondheim, Norway; 3 Department of Biotechnology and Nanomedicine, SINTEF Industry, 7465 Trondheim, Norway

**Keywords:** Microbioreactor, Fermentation, Lignocellulosic sugar, Butanol, *Clostridium*, ANOVA

## Abstract

**Background:**

Butanol (*n-*butanol) has been gaining attention as a renewable energy carrier and an alternative biofuel with superior properties to the most widely used ethanol. We performed 48 anaerobic fermentations simultaneously with glucose and xylose as representative lignocellulosic sugars by *Clostridium beijerinckii* NCIMB 8052 in BioLector® microbioreactors to understand the effect of different sugar mixtures on fermentation and to demonstrate the applicability of the micro-cultivation system for high-throughput anaerobic cultivation studies. We then compared the results to those of similar cultures in serum flasks to provide insight into different setups and measurement methods.

**Results:**

ANOVA results showed that the glucose-to-xylose ratio affects both growth and production due to *Carbon Catabolite Repression*. The study demonstrated successful use of BioLector® system for the first time for screening several media and sugar compositions under anaerobic conditions by using online monitoring of cell mass and pH in real-time and at unprecedented time-resolution. Fermentation products possibly interfered with dissolved oxygen (DO) measurements, which require a careful interpretation of DO monitoring results.

**Conclusions:**

The statistical approach to evaluate the microbioreactor setup, and information obtained in this study will support further research in bioreactor and bioprocess design, which are very important aspects of industrial fermentations of lignocellulosic biomass.

## Background

Renewable chemicals and fuels have gained interest worldwide as a result of increasing global warming and climate change concerns, volatility of oil price and supply as well as legal restrictions on nonrenewable energy sources [1]. Driven by these motivations, global actors have come up with goals to increase the share of renewables and scenarios to predict the future energy mix. For example, the European Commission planned to replace 25% of traditional fuels with biofuels by 2030, and the International Energy Agency foresees an increase of 25% by 2024 compared to the global biofuel production of 10 billion liters in 2018 [2].

*n-*Butanol (in the following simplified as butanol) has been gaining attention as a renewable energy carrier for biofuel applications with superior properties such as a higher energy density of 29.2 MJ/l compared to that of ethanol (19.6 MJ/l) and methanol (16 MJ/l), and a lower heat of vaporization (0.43 MJ/kg) than that of ethanol (0.92 MJ/kg) and methanol (1.2 MJ/kg) which provides an easier engine start [3]. Moreover, butanol run engines have lesser ignition problems due to a lower autoignition temperature of 385 °C compared to 434 °C and 470 °C for ethanol and methanol, respectively [3, 4]. In addition, diesel engines can run on pure butanol or diesel blends without any modifications and apparent damage [5]. Based on a recent report from Reuters (April 2019), the butanol market will register a 3.5% CAGR (compound annual growth rate) in terms of revenue over the next 5 years (2019–2024), and the global market size will reach US$ 7.7 billion by 2024, from US$ 6.4 billion in 2019 [6]. The majority of butanol is produced via petrochemical reaction; the propylene hydroformylation, also known as oxo route [7, 8], creating a close link to the propylene market, thus to the price of crude oil [8]. Therefore, butanol production via the petrochemical route is not favorable due to environmental concerns as mentioned above, creating a greater interest in fermentative butanol production. Despite these driving factors, fermentative butanol production still faces multiple challenges such as feedstock availability, costly product recovery, and low product yield as discussed thoroughly in our literature review article [9]. These issues need to be addressed with expanded research and development in order to render butanol production at large scale economically viable.

To ensure broad feedstock availability, lignocellulosic biomass is targeted widely since it is the most abundant renewable energy resource on the planet and avoids the direct fuel-versus-food competition caused by using, e.g., corn and sugar cane in biofuel production. The composition of lignocellulose depends on the plant species, age and growth conditions with typical dry weight compositions of 34.2–46.4% glucose, 4.9–24.9% xylose, 1.1–2.9% arabinose, 0.3–12% mannose, and 11.9–29.4% lignin as reported in literature [10]. Therefore, hydrolysis of lignocellulosic polysaccharides yields a mixture of C5 and C6 sugars, which can be fermented to butanol typically by bacteria of the genus *Clostridium*. Current methodologies still mainly focus the fermentation of glucose while discarding the rest of the feedstock or using it as a source of process energy. However, the complete exploitation of all the sugars, particularly the major C5 sugar xylose bound in lignocellulosic biomass can contribute to solving the low yield challenge that is one of the main issues related to fermentative butanol production.

The cells’ efficiency at metabolizing different sugars in mixed form tends to be limited by a phenomenon called *Carbon Catabolite Repression* (CCR). CCR reduces or prevents the utilization of C5 sugars in the presence of a preferred carbon source such as the C6 sugar glucose [11]. In our previous work, we investigated the effects of CCR on the utilization of lignocellulosic sugars in mixed form [12], modeled the cell growth on mixed sugars in a follow-up study [13] and in a later study, extended the model with sugar consumption and butanol production [14]. In the present study, we performed lignocellulosic sugar fermentations in microbioreactors and compared them to similar cultures in serum flasks for validating our previous findings, as an important step in paving the way for a more efficient, systematic bioprocess development in second generation butanol production.

Systematic bioprocess development involving strain cultivation, optimization, and testing is often needed to increase yield and productivity. These efforts require screening of strains, medium compositions, and operating conditions, which are traditionally carried out in shake flasks or microtiter plates. However, these methods have some downsides, such as the lack of online monitoring and control [15] as well as automation requirement for easy handling of increased number of experiments [16]. Microbioreactor technology can eliminate some of these drawbacks by offering easy handling, online monitoring of key parameters and control capability, in addition to the possibility to run multiple cultures in parallel. Disposable and miniaturized versions of bench-scale bioreactors are today available for performing fermentation experiments. Moreover, such technology has the advantage of low power consumption, less space requirements, small quantities of reagents and cells per batch as well as flexibility and portability due their small size [17]. To exploit these advantages for studying fermentative butanol production on mixed sugars derivable from lignocellulosic biomass, we performed clostridial fermentation experiments in BioLector® microbioreactors. The BioLector® instrument (m2p-labs GmbH, Baesweiler, Germany) is a powerful tool with proven capabilities of high-throughput fermentation with simultaneous online monitoring of cell mass (by light scattering), fluorescence, pH and dissolved oxygen (DO) [18]. To our knowledge, the BioLector® has not been used for the purpose of studying fermentative butanol production before. Thus, the main objective of this study is to show the effect of different sugar mixtures on growth kinetics and butanol production together with the first general demonstration of the use of BioLector® for butanol production under anaerobic conditions in comparison to the widely used serum flasks.

## Results

### Monitoring of growth in BioLector® microbioreactor fermentations

The growth, utilization of carbon source and butanol production was measured using 12 different conditions, namely the amount (5 or 10 g/l) and mixture [ratio between glucose (G) and xylose (X)] of C-sources. All glucose contained in the fermentation medium was utilized entirely in all 12 conditions of the fermentation experiment (79 h). On the other hand, there was residual xylose in 5 of 12 experiments which are 10-G80:X20, 10-G40:X60, 10-G20:X80, 10-G0:X100 and 5-G0:X100 with xylose amounts of 0.040, 0.037, 0.060, 0.086 and 0.014 g/l, respectively.

**Fig. 1:**
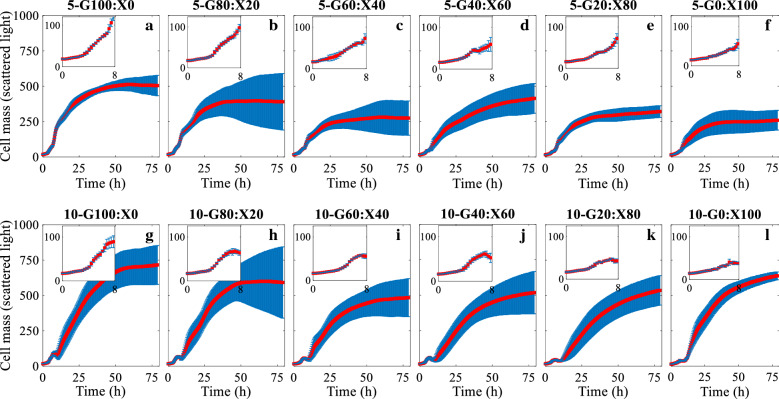
Cell mass versus time plots of fermentations done in BioLector® using 5 and 10 g/l total sugar and varied glucose (G)-to-xylose (X) ratios. **a** 5-G100:X0, **b** 5-G80:X20, **c** 5-G60:X40, **d** 5-G40:X60, **e** 5-G20:X80, **f** 5-G0:X100, and **g** 10-G100:X0, **h** 10-G80:X20, **i** 10-G60:X40, **j** 10-G40:X60, **k** 10-G20:X80, and **l** 10-G0:X100. Mean values of the 4 replicas are shown together with error bars representing standard deviation, and the magnified parts of each subplot are placed on top left corner

**Fig. 2:**
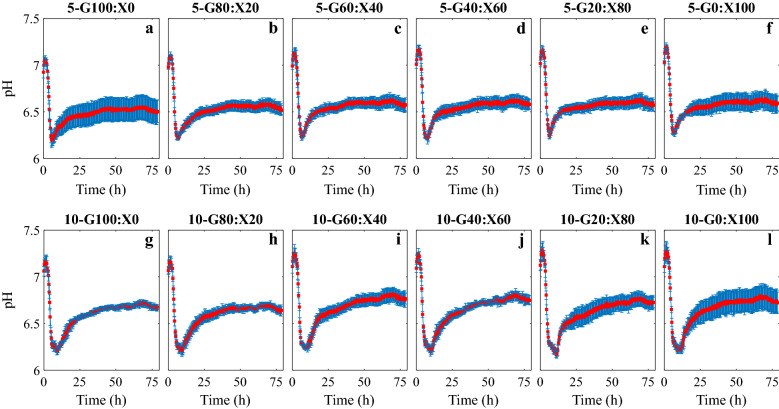
pH versus time plots of fermentations done in BioLector*®* using 5 and 10 g/l total sugar and varied glucose (G)-to-xylose (X) ratios. **a** 5-G100:X0, **b** 5-G80:X20, **c** 5-G60:X40, **d** 5-G40:X60, **e** 5-G20:X80, **f** 5-G0:X100, and **g** 10-G100:X0, **h** 10-G80:X20, **i** 10-G60:X40, **j** 10-G40:X60, **k** 10-G20:X80, and **l** 10-G0:X100. Mean values of the 4 replicas are shown together with error bars representing standard deviation

**Fig. 3:**
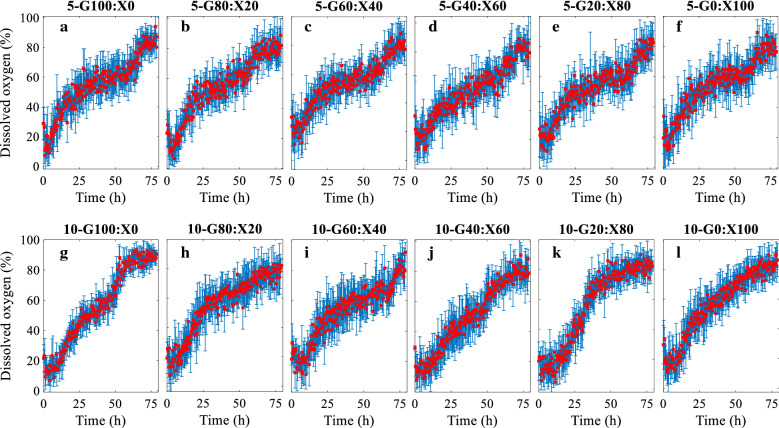
Dissolved oxygen (%) versus time plots of fermentations done in BioLector*®* using 5 and 10 g/l total sugar and varied glucose (G)-to-xylose (X) ratios. **a** 5-G100:X0, **b** 5-G80:X20, **c** 5-G60:X40, **d** 5-G40:X60, **e** 5-G20:X80, **f** 5-G0:X100, and **g** 10-G100:X0, **h** 10-G80:X20, **i** 10-G60:X40, **j** 10-G40:X60, **k** 10-G20:X80, and **l** 10-G0:X100. Mean values of the 4 replicas are shown together with error bars representing standard deviation

Figures 1, 2, 3 present online logged data of the fermentations done in BioLector® in terms of cell mass (scattered light), pH and DO (%) allowing the continuous monitoring of growth and acid production and utilization.

Right after inoculation, we observed a lag phase of approximately 1.5 h and 1 h for cultures containing 5 and 10 g/l total sugar, respectively (Fig. 1), likely due to the adaptation of the cells to their new environment [19]. After the lag phase, exponential growth was observed for several hours, after which a phase of continuously decreasing growth rate occurred until stationary phase was reached.

The pH of the fermentation broth (Fig. 2) decreased due to the production of acetic acid and butyric acid during the exponential growth phase [20]. pH subsequently increased again during the phase of decreasing growth rate as the produced acids were re-assimilated to form solvents. The experiments under anaerobic conditions were repeated under same conditions and they all exhibited increasing DO levels as the growth proceeded. The start of the experiments was successful in terms of achieving anaerobic conditions in all runs; however, an increase of DO was observed during the experiments (Fig. 3). Standard deviations were considerably smaller during lag and exponential growth phases compared to the rest of the fermentation for both cell mass and pH values while there was no apparent correlation with time for DO.

### Correlation of cell mass and dissolved oxygen in BioLector® microbioreactor fermentations

**Fig. 4:**
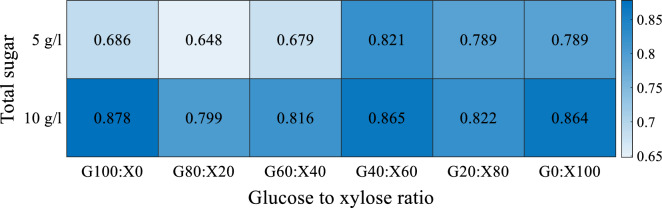
Correlations between cell mass and dissolved oxygen for fermentations done in BioLector®

Kendall’s tau correlation coefficient was used to determine the correlations between cell mass (scattering light) and DO (%) because it is able predict nonlinear relationships [21] and robust in presence of outliers in data [22]. The correlations are shown in Fig. 4 for all experiments.

The coefficient has a value between + 1 and − 1, where 1 is total positive correlation, 0 is no correlation, and − 1 is total negative correlation. When the absolute value of the correlation coefficient is smaller than 0.35, the correlation is considered to be weak; for values between 0.36 and 0.67, the correlation can be regarded as moderate; a correlation is strong for coefficient values greater than 0.68 [23].

As can be seen in Fig. 4, correlations between cell mass and DO were positive and strong for all 12 experiments with no apparent relation with the glucose-to-xylose ratio or total sugar concentration.

**Fig. 5:**
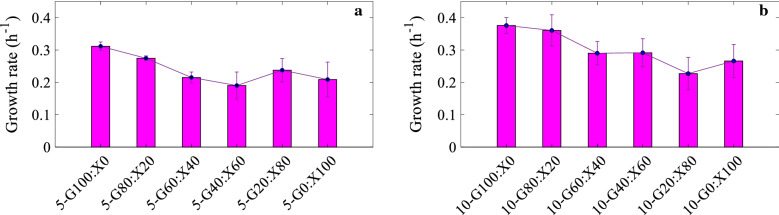
Growth rate (h^−1^) values during exponential growth for all 12 experiments done in the BioLector® setup were determined from the BioLector® online data

Figure 5 shows that growth rate (h^−1^) values were higher for cultures with 10 g/l total sugar. Average growth rate values for cultures containing 5 and 10 g/l were 0.240 and 0.302 h^−1^, respectively. However, the change in growth rate with respect to glucose-to-xylose ratio exhibited the opposite trend for ratios of G100:X0, G80:X20 and G60:X40. Highest and lowest growth rates were 0.376 and 0.190 h^−1^ observed for 10-G100:X0 and 5-G40:X60 cultures.

### Comparison of BioLector® microbioreactor and serum flask fermentations

**Fig. 6:**
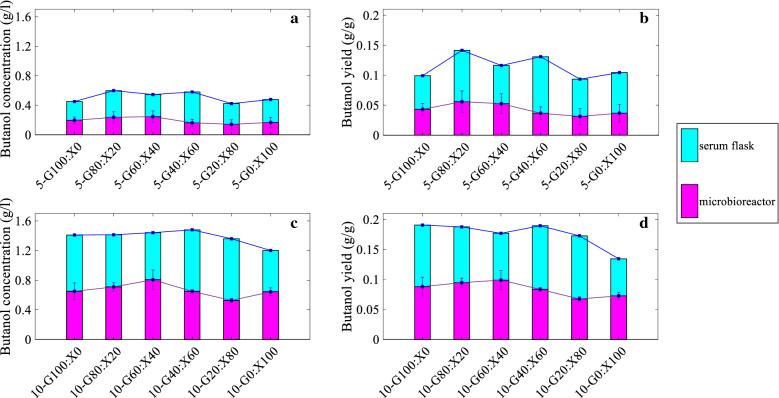
Butanol concentration **a** and **c**, and butanol yield **b** and **d** values of fermentations done in BioLector® and serum flasks with 5 and 10 g/l total sugar, respectively. Mean values of the 4 replicas are shown together with error bars representing standard deviation for BioLector® results

A comparative overview of the results for serum flask and microbioreactor setups is provided in this section. Both glucose and xylose contained in the fermentation medium were utilized entirely in all 12 conditions in serum flasks, while there was some residual xylose in microbioreactor experiments as given in the results above. Butanol concentration (g/l) and butanol yield (g butanol/g sugar) values for all are summarized in Fig. 6.

Figure 6 shows that butanol concentrations and yields were higher for cultures containing 10 g/l total sugar for all 6 different glucose-to-xylose ratios in both setups. For the BioLector®, the average butanol concentration and butanol yield values of 6 experiments with 5 and 10 g/l were 0.192 and 0.664 g/l, 0.043 and 0.084 g/g, respectively. Both butanol concentrations and yields increased with increasing xylose ratios from 0 to 40, decreased when xylose ratio increased from 40 to 80, and increased again when the ratio was G0:100X for cultures with 5 and 10 g/l total sugar. Highest butanol concentration and butanol yield were 0.806 g/l and 0.099 g/g achieved in the 10-G60:X40 culture, while lowest values 0.142 g/l and 0.031 g/g were observed in 5-G20:X80.

For the serum flask setup, average butanol concentration and butanol yield values of 6 the experiments each with 5 and 10 g/l sugars were 0.513 and 1.384 g/l, and 0.114 and 0.176 g/g, respectively. Thus, higher total sugar resulted in a higher butanol production and yields, which is in good agreement with the results obtained in BioLector® fermentations. Highest butanol concentration and butanol yield were 1.480 g/l and 0.190 g/g, achieved in the 10-G40:X60 culture, while lowest values were 0.422 g/l and 0.093 g/g observed in 5-G20:G80, which coincide with results of BioLector® as well.

We performed a two-way ANOVA to assess if the effect of changing total sugar concentrations and glucose-to-xylose ratios on fermentation were significant. Two tests were performed for fermentations done in BioLector® and in serum flasks, which are summarized in Tables 1, 2.

**Table 1:** *p* values obtained from ANOVA for fermentations done in BioLector® to assess the effect of total sugar concentration and sugar ratio on butanol concentration, butanol yield and specific growth rate

	Butanol concentration (g/l)	Butanol yield (g/g)	Specific growth rate (h^−1^)
Total sugar concentration (g/l)	1.54E−08	1.54E−08	3.71E−05
Sugar ratio (g/g)	0.00063	0.00025	2.88E−05

**Table 2:** *p* values obtained from ANOVA for fermentations done in serum flasks to assess the effect of total sugar concentration and sugar ratio on butanol concentration and butanol yield

	Butanol concentration (g/l)	Butanol yield (g/g)
Total sugar concentration (g/l)	0.01431	0.01431
Sugar ratio (g/g)	0.14118	0.2794

Table 1 shows that both total sugar concentration (g/l) and glucose-to-xylose sugar ratio have significant effect on butanol concentration (g/l), butanol yield (g butanol/g sugar) and growth rate (h^−1^), since all *p* values are smaller than 0.05. It is important to note that the effect of total sugar concentration on butanol concentration (g/l) and butanol yield (g butanol/g sugar) was greater than that of sugar ratio with *p* values of 1.54E−08 and 1.54E−08, and 0.00063 and 0.00025, respectively. On the other hand, significance of effects for total sugar concentration and sugar ratio was very similar for specific growth rate with *p* values of 3.71E−05 and 2.88E−05, respectively. Therefore, the specific growth rate was equally sensitive to both factors.

Table 2 shows that the effects of total sugar concentration on butanol concentration and butanol yield were significant with *p* values smaller than 0.05, while the sugar ratio did not have a significant effect. The effect of total sugar concentration was less pronounced in serum flasks than what was observed in the BioLector® setup.

## Discussion

For the best understanding of carbon turnover and product formation, fermentation progress in standard bioreactors is followed continuously by online measurements of pH, DO, and off-gas CO_2_, while cell mass, sugar and product concentrations are usually determined intermittently by offline spectrophotometry and HPLC, respectively. Such experiments are, however, laborious and costly, and the first phase of process optimization, involving, e.g., different parameters and strains, is therefore often performed in serum flasks. We used, to our knowledge for the first time, the BioLector® technology to perform 48 anaerobic fermentations with real-time monitoring of cell mass and pH to establish it as a potential alternative to serum flask cultivations. The cell mass monitoring allowed the direct calculation of growth rates during cultivations, whereas the pH measurements provided online information about sugar consumption and switching of clostridial metabolism from acetogenic to solventogenic phase. This represents an improvement over standard offline measurement methods in the evaluation of clostridial fermentations, even though off-gas CO_2_, sugars and product concentrations are still not measured online.

**Fig. 7:**
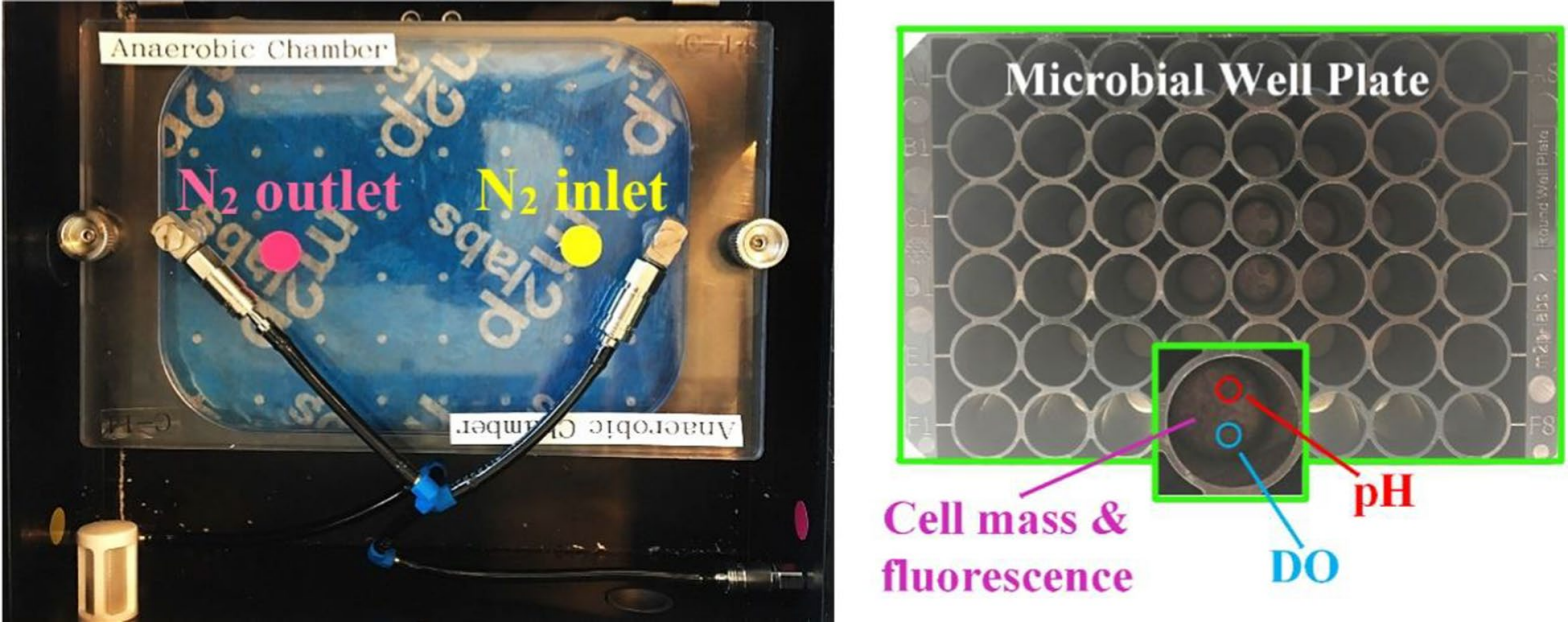
BioLector® anaerobic chamber (left), round-well plate, and a single well showing the sensors for dissolved oxygen (DO) and pH, and the area for detecting cell mass by means of light scattering and fluorescence (right)

The fermentation experiments under anaerobic conditions were repeated under same conditions and they all exhibited increasing DO levels as the growth proceeded. The start of the experiments was successful in terms of achieving anaerobic conditions in all runs; however, an increase was observed during the experiments. This observation can be explained by (i) a problem with the measurement and/or sensor and (ii) diffusion/leakage of O_2_ from the outside environment to the BioLector® chamber. The latter is prevented by use of a protective layer on the well plate as well as a sealing of the well plate in the anaerobic chamber screwed tightly where only openings are the gas (N_2_) inlet and outlet (Fig. 7). Moreover, *C. beijerinckii* are strict anaerobes which cannot grow in presence of oxygen, and they produce CO_2_ and H_2_ as they grow that also helps to maintain anaerobic conditions in the growth medium [24]. Therefore, it is more likely to be the first reason as evident also from the correlation analysis results; strong and positive correlations between cell mass and DO were found for all experiments, which is in line with the previous observations obtained from the study of online monitoring for high-throughput screening of microbial systems in the BioLector® system [25]. It is also important to note that CO_2_ is not possible to be measured in BioLector® even though it gives a good indication for the growth status. Therefore, interference of CO_2_ with DO measurement should be investigated in future studies.

Understanding the effect of the glucose-to-xylose ratio in substrate mixtures is necessary for successful design of efficient lignocellulosic biomass fermentations. For that reason, we first studied the effect of sugar ratio on cell mass growth rate. For experiments done in BioLector®, all cultures containing xylose had a slower growth rate than the cultures with glucose as the sole sugar, essentially due to glucose being the preferred carbon source over xylose and the effect of CCR [11]. Therefore, our observation confirms that cell mass growth was affected by CCR during the fermentation by *C. beijerinckii* NCIMB 8052. The growth rate decreased with decreasing glucose ratio in the medium. However, when xylose was the sole sugar (G0:X100), the growth rate was similar to that of G20:X80 cultures. This trend can be assigned to noncompetitive interaction between sugars and is consistent with the results in our earlier study [13]. The standard deviation values of cell mass data increased significantly in stationary phase compared to exponential growth phase as shown in Fig. 1. Different glucose-to-xylose ratios resulted in different standard deviations as well. The reason may be accounted to changes in the morphology of cells affecting the online cell mass monitoring in the BioLector® unit [25], since Clostridia are known to go through morphological changes during fermentation [26], and variation of standard deviation might indicate that the sugar composition affects the morphology of the cells. The same trend was observed for online logged pH data; standard deviations were greater when the cultures reached stationary phase. We investigated if any particular replicas deviated from the mean value due to its location on the well plate; however, no obvious correlation was obtained, and further systematic examination is required to rule out potential position effects. The sugar ratio also had impact on butanol concentration and yield, which were inversely proportional with the growth rate. Our results are in accordance with a previous study, which showed that a higher growth rate results in a lower butanol concentration, since sugars were used for cell mass growth and not for butanol production [27].

Comparison of fermentations performed in microbioreactors and serum flasks can provide important insight for use of different bioreactors and monitoring methods. Growth rates estimated using online logged cell mass data of BioLector® were in the range of 0.190–0.312 h^−1^ for the cultures with 5 g/l total sugar, which is significantly lower than the range of 0.681–1.076 h^−1^ obtained in our previous study done in serum flasks using the same sugar concentration and strain [12]. Similarly, the average growth rate of 0.240 h^−1^ in microbioreactors was 70.7% lower than the value of 0.819 h^−1^ acquired in serum flasks. Different growth rate values obtained in microbioreactors and serum flasks may be explained by the difference in cell mass measurement methods. A study comparing shake flasks and BioLector® microbioreactors showed that cell mass measurements in BioLector® (scattered light intensity) and measurements with photometer (optical density) were in good agreement for the growths of *E. coli* and *K. lactic*. Contrarily, cell mass values for growth of *G. oxydans* differed greatly, which was explained by morphological changes [28]. Recent work of Petra et al. showed the changing morphology throughout the life cycle of *Clostridium beijerinckii* [29]. Therefore, it is important to have good knowledge about the physiology of the strain used when comparing different experimental setups with different monitoring technologies.

The effect of sugar ratio on butanol concentration and yield was not as distinguishable for fermentations done in serum flasks as for the microbioreactor fermentations. However, highest butanol productions and yields were observed in the 10-G60:X40 and 10-G40:X60 cultures, while lowest values were observed in the 5-G20:X80 cultures in both experimental setups. Average butanol concentration and butanol yield values for all 12 experiments done in microbioreactors were 0.428 g/l and 0.063 g/g being 54.9 and 56.6% lower than in serum flask fermentations with average values of 0.948 g/l and 0.145 g/g. Even though butanol concentrations were low due to low sugar concentrations used in the fermentations of this study, the average value of butanol yield obtained in serum flasks is comparable with the butanol yield found as 0.198 g/g in our exploratory data analysis performed by using data of 79 fermentations with lignocellulosic sugars [9]. The agreement in butanol yield values is noteworthy, since it is a measure of the cells’ efficiency to convert substrate into the desired product. Even though same inoculated media were used in microbioreactors and serum flasks to have identical starting conditions, there were significant deviations in butanol concentration and butanol yield values, which can be related to the differences in experimental setups. Microbial culture wells in the microbioreactor setup were continuously shaken and flushed with nitrogen to ensure and anaerobic conditions. This might have caused a stripping effect for the volatile components present in fermentation broth, since gas stripping is a commonly applied method for butanol removal [30]. Although an evaporation-limiting layer was used, it might still be permeable to butanol fume. The serum flask fermentations were performed under static conditions without any gas flow through the flasks. Moreover, flasks were sealed with rubber stoppers to sustain anaerobic conditions. Therefore, the gas stripping effect was not as pronounced as in microbioreactors, which could explain the determined higher average butanol concentration, thus average butanol yield.

Experimental observations were further analyzed by using ANOVA tests, and the results showed that both total sugar concentration and glucose-to-xylose ratio had significant effects on the fermentations done in BioLector® with specific growth rate being the most sensitive. ANOVA also showed that the effect of sugar ratio was not profound for fermentations done in serum flasks. The difference in ANOVA results for the two different experimental setups confirmed their different impact on fermentation. An in-depth metabolic study would be useful to investigate the effects of different carbon sources on *C. beijerinckii* NCIMB 8052.

## Conclusions

We performed fermentations of glucose and xylose at 6 different ratios and 2 different total sugar concentrations by *Clostridium beijerinckii* NCIMB 8052 in microbioreactors and serum flasks to show the effect of different sugar mixtures on growth kinetics and butanol production and to demonstrate the use of BioLector® for fermentative butanol production under anaerobic conditions. Main findings of this study and their significance are summarized as follows:

- The results showed that the glucose-to-xylose ratio affects both growth and production due to CCR, which might enable control of both by optimizing the sugar composition, thus successful design, and operation of efficient lignocellulosic biomass fermentations.
- All cultures grew successfully in the BioLector® system under anaerobic conditions, metabolized both glucose and xylose as representative lignocellulosic sugars, and produced butanol.
- The online monitoring of cell mass and pH enabled us to follow the progress of the fermentation in real-time and at unprecedented time-resolution.
- The online monitoring of DO values should be treated with more attention due to its possible interference with other fermentation product to avoid any misinterpretations.
- Demonstrating a successful use case of BioLector® for fermentative butanol production provides know-how to the scientific community that can enable more informed decisions for the design of the experiments as well as for the selection of the technology.
- BioLector® system is well suited for anaerobic screening of several media and sugar compositions prior to selection of a few conditions for either serum flask experiments or the more laborious laboratory-scale fermentations with full online and offline monitoring.

In conclusion, the information obtained in this study will support further research in bioreactor and bioprocess design, which are very important aspects of industrial fermentations of lignocellulosic biomass. In future studies, fermentation of lignocellulosic biomass hydrolysates can be performed in BioLector® to advance the knowledge in the field and to exploit the fast and efficient screening advantages of the setup.

## Materials and methods

### Microorganism and medium

*Clostridium beijerinckii* NCIMB 8052 was used in this study, since it is known to utilize different lignocellulosic sugars for growth and butanol production [31]. First, a frozen work ampoule was pre-grown for 14 h on 50 ml of reinforced clostridial medium (CM0149, Oxoid) in an incubator at 37 °C under anaerobic and static conditions. This pre-grown culture was used as inoculum for all experiments (both microbioreactors and serum flasks). The fermentation medium composition was 5 or 10 g/l sugar (different ratios of xylose and glucose as explained later in this section), 2.5 g/l Na-acetate, 5 g/l yeast extract, 2 g/l (NH_4_)_2_SO_4_, 0.01 g/l NaCl, 0.75 g/l KH_2_PO_4_, 1.5 g/l K_2_HPO_4_, 0.2 g/l MgSO_4_.7H_2_O, 0.01 g/l MnSO_4_.H_2_O, 0.01 g/l FeSO_4_.7H_2_O, 0.01 g/l *P*-aminobenzoic acid, 0.01 g/l biotin and 0.1 g/l thiamine. Six different mixed sugar solutions of glucose and xylose were prepared at 6 different ratios with a total sugar concentration of 5 and 10 g/l. Consequently, in total 12 different sugar compositions using 2 different total sugar concentrations and 6 different glucose-to-xylose ratios were studied in parallel. The cultures containing 5 g/l total sugar, and glucose (G)-to-xylose (X) ratios of 100:0, 80:20, 60:40, 40:60, 20:80 and 0:100 are referred to as 5-G100:X0, 5-G80:X20, 5-G60:X40, 5-G40:X60, 5-G20:X80 and 5-G0:X100, respectively, throughout the study. Similarly, the cultures containing 10 g/l total sugar and glucose-to-xylose ratios of 100:0, 80:20, 60:40, 40:60, 20:80 and 0:100 are referred to as 10-G100:X0, 10-G80:X20, 10-G60:X40, 10-G40:X60, 10-G20:X80 and 10-G0:X100, respectively.

### Fermentations

Fermentations were performed in in batch mode in round-well plates with 48 × 3 ml microbioreactor wells with 1.5 ml working volume for 79 h. Anaerobic conditions were sustained by flushing with nitrogen gas (Aga grade 6.0) at 37 °C and shaking at 400 rpm in a BioLector® instrument (m2p-labs GmbH, Baesweiler, Germany) that was kept constant throughout the whole fermentation. Figure 7 shows the BioLector® anaerobic chamber together with a round-well plate and a single well with embedded sensors.

Cultures in the wells were started by adding 4% (v/v) inoculum prepared as described above. The BioLector® measures cell mass density by scattered light, applied in the present study in 20 min intervals. A gain of 20 (EX: 620 nm, EM: 620 nm) was used for the experiments to avoid saturation at high cell mass. pH was measured every 20 min with a gain of 19 (EX: 470 nm, EM: 525 nm).

**Fig. 8:**
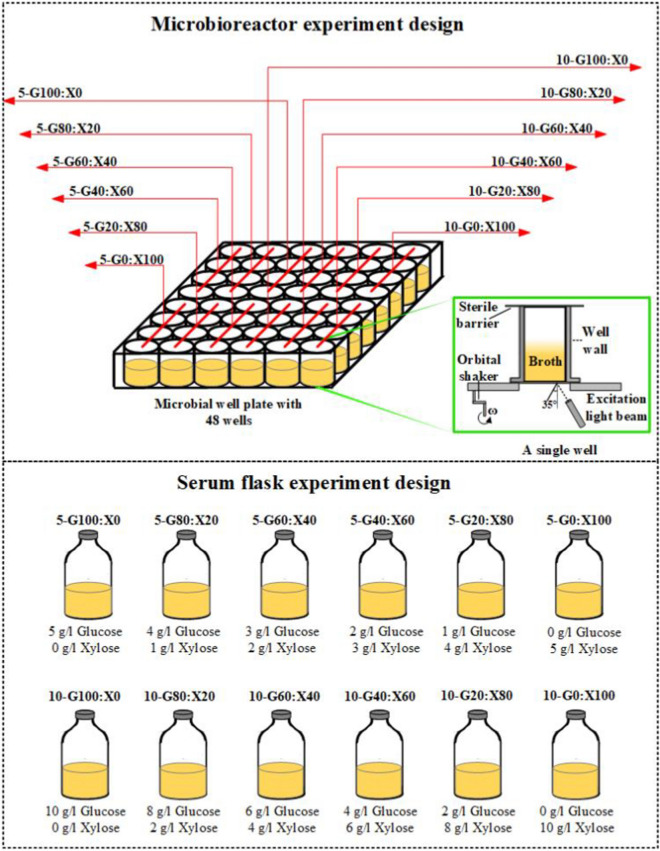
Experimental design of fermentations performed in microbioreactors with a schematic representation of a single well (top) and serum flasks (bottom)

To benchmark the microbioreactor fermentations, fermentations in 120-ml serum flasks were performed with 50 ml working volume in an incubator at 37 °C under static and anaerobic conditions, with 12 different sugar compositions as explained above. A representation of the experimental design of fermentations performed both in microbioreactors and serum flasks is shown in Fig. 8.

Both the microbioreactor and the serum flask experiments were performed using the same batch of medium and inoculum to minimize errors due to medium preparation and inoculation. Inoculum size was 4% (v/v) in all cases. There was no pH control applied. Experiments were terminated after 79 h. Samples were taken at the start and the end of the fermentations for analysis of medium components and products, cell mass and pH. Data shown represent the mean values from experiments performed in quadruples, and error bars represent the standard deviations in microbioreactor results.

### HPLC

After cultivation, fermentation samples from both microbioreactors and serum flasks were used to determine residual sugars and fermentation products by high-performance liquid chromatography (HPLC). The samples were filtrated (Millipore filter, 0.2 µm) before HPLC analysis on an Agilent System LC1260 equipped with UV (210 nm) and RI detector and an Aminex HPX-87H column (BioRad). Samples were eluted with 5 mM H_2_SO_4_ at a flow rate of 0.6 ml/min at 45 °C. Quantification was performed using standards for each component.

### Estimation of kinetic coefficients

Cell mass growth rates were estimated during exponential growth phase in which nutrients are non-limiting and thus the growth rate is independent of the nutrient concentration. Therefore, the rate of growth is

$$\frac{{\text{d}}X}{{\text{d}}t}=\mu X$$

where *X* is the cell mass concentration (g/l), t is time (h), and *µ* is the specific growth rate (h^−1^). The specific growth rate is determined by estimating the slope of the cell mass concentration versus time plot.

The product yield based on substrate consumption is a commonly used kinetic coefficient that indicates how efficient the conversion of substrate to product of interest is [32]. The product yield (g product/g sugar) is

$$YP/S=\frac{\Delta{P}}{\Delta{S}}$$

where *P* is the product concentration, butanol (g/l), and *S* is the total substrate concentration, glucose and xylose (g/l).

### Kendall's tau correlation coefficient

Kendall's correlation coefficient shows the correlations among pairs of variables in a data set. Matlab function, corr is used for this purpose with pairwise option so that correlation coefficient is computed only for the rows with no missing values in columns *i* or *j*. Kendall’s tau is based on counting the number of (*i*,*j*) pairs, for *i *< *j*, that are concordant. That is for which *X*_*a*,*i*_ – *X*_*a*,*j*_ and *Y*_*b*,*i*_ – *Y*_*b*,*j*_ have the same sign. The equation for Kendall’s tau includes an adjustment for ties in the normalizing constant. For column *X*_*a*_ in matrix *X* and column *Y*_*b*_ in matrix *Y*, Kendall's tau correlation coefficient is

$$\tau =\frac{2K}{n(n-1)}$$

where $$K={\sum }_{i=1}^{n-1}{\sum }_{j=i+1}^{n}{\xi }^{*}({X}_{a,i},{X}_{a,j},{Y}_{b,i},{Y}_{b,j})$$ and

$${\xi }^{*}({X}_{a,i},{X}_{a,j},{Y}_{b,i},{Y}_{b,j})=\left\{\begin{array}{c}1 if {(X}_{a,i}-{X}_{a,j})({Y}_{b,i}{-Y}_{b,j})>0\\ 0 if {(X}_{a,i}-{X}_{a,j})(Y_b,i-Y_b,j)=0\\ -1 if {(X}_{a,i}-{X}_{a,j})({Y}_{b,i}{-Y}_{b,j})<0\end{array}\right.$$

### ANOVA

Analysis of variance (ANOVA) is a procedure to assign sample variance to different sources and to decide whether the variation arises within or among different population groups. A two-way, nonparametric ANOVA method, Friedman's test is used in this study [33]. The *p* value that the Friedman's test returns is used to determine significance. If the *p* value is near zero, this casts doubt on the null hypothesis. A sufficiently small *p* value suggests that at least one column-sample median is significantly different from the others. It is common to declare a result significant if the *p* value is less than 0.05 or 0.01, and we chose 0.05 in this study. Matlab function, friedman is used with replicate number of 4 for fermentations done in BioLector® and 1 for fermentations done in serum flasks. Two different effects are considered: total sugar concentration values of 5 and 10 g/l, and glucose-to-xylose ratios of 100:0, 80:20, 60:40, 40:60, 20:80, 0:100.

## Data Availability

All data and materials used in this study are available upon request.

## Abbreviations

ANOVA: Analysis of variance
CCR: Carbon catabolite repression
DO: Dissolved oxygen
HPLC: High-performance liquid chromatography
